# Supplementary material for: Prognostic role of interim F-18 fluorodeoxyglucose positron emission tomography-computed tomography during chemoradiation therapy in patients with hypopharyngeal squamous cell carcinoma
Source: PLoS One. 2026 Feb 5;21(2):e0341189. doi: 10.1371/journal.pone.0341189 (PMC12875515; doi:10.1371/journal.pone.0341189)

PLOS ONE

Prognostic role of interim F-18 fluorodeoxyglucose positron emission tomography-computed tomography during chemoradiation therapy in patients with hypopharyngeal squamous cell carcinoma

--Manuscript Draft--

**Manuscript Number:**	PONE-D-25-48920
**Article Type:**	Research Article
**Full Title:**	Prognostic role of interim F-18 fluorodeoxyglucose positron emission tomography-computed tomography during chemoradiation therapy in patients with hypopharyngeal squamous cell carcinoma
**Short Title:**	Prognostic role of interim FDG PET/CT during chemoradiation in hypopharyngeal cancer
**Corresponding Author:**	Yoshihito Nomoto<br>Mie University: Mie Daigaku<br>Tsu, Mie JAPAN
**Keywords:**	Hypopharyngeal squamous cell carcinoma<br>Chemoradiotherapy<br>Positron emission tomography/computed tomography<br>Prognostic indicator
**Abstract:**	The aim of this study was to investigate the usefulness of interim F-18 fluorodeoxyglucose positron emission tomography-computed tomography (FDG PET/CT) during definitive radiation therapy as a prognostic indicator of disease recurrence in patients with hypopharyngeal squamous cell carcinoma. This prospective analysis included 35 patients newly diagnosed with hypopharyngeal squamous cell carcinoma who received platinum-based chemoradiation therapy and underwent pretreatment FDG PET/CT (prePET) and interim FDG PET/CT (iPET) at cumulative RT doses of 36.0–45.0 Gy. The maximum standardized uptake value (SUVmax), metabolic tumor volume, and total lesion glycolysis of the primary tumor and combined total lymph nodes for both prePET and iPET were analyzed, and their percentage reductions in iPET were calculated. The optimal cutoff values of metabolic parameters were derived from receiver operating characteristic curves. Outcomes were compared between patients with metabolic parameters above/below the respective cutoff values. Twelve (34%) patients exhibited disease recurrence during a median follow-up of 4.3 years. Univariate Cox regression analysis revealed that the reduction ratio of the SUVmax of the primary tumor (ΔSUVp; hazard ratio, 7.685; p = 0.008) was a significant predictor of disease recurrence. Kaplan–Meier curve analysis revealed that a smaller ΔSUVp was associated with worse progression-free survival (log-rank, p = 0.002). Metabolic parameters measured via iPET may be useful predictors of disease recurrence in patients with hypopharyngeal squamous cell carcinoma treated with chemoradiation therapy. In this study, ΔSUVp was the best such prognostic indicator.
**Order of Authors:**	Takamitsu Mase
	Yutaka Toyomasu
	Hajime Ishinaga
	Yui Nanpei
	Tomoko Kawamura
	Akinori Takada
	Yasutaka Ichikawa
	Noriko Ii
	Tomoya Hirata
	Kazuhiko Takeuchi
	Hajime Sakuma

Powered by Editorial Manager® and ProduXion Manager® from Aries Systems Corporation

	Yoshihito Nomoto
**Additional Information:**	
**Question**	**Response**
**Financial Disclosure**<br><br>Enter a financial disclosure statement that describes the sources of funding for the work included in this submission. Review the submission guidelines for detailed requirements. View published research articles from *PLOS ONE* for specific examples.<br><br>This statement is required for submission and **will appear in the published article** if the submission is accepted. Please make sure it is accurate.<br><br>**Funded studies**<br>Enter a statement with the following details:<br>• Initials of the authors who received each award<br>• Grant numbers awarded to each author<br>• The full name of each funder<br>• URL of each funder website<br>• Did the sponsors or funders play any role in the study design, data collection and analysis, decision to publish, or preparation of the manuscript?<br><br>Did you receive funding for this work?	The author(s) received no specific funding for this work.
**Competing Interests**<br><br>Use the instructions below to enter a competing interest statement for this submission. On behalf of all authors, disclose any competing interests that could be perceived to bias this work—acknowledging all financial support and any other relevant financial or non-financial competing interests.<br><br>This statement is required for submission and **will appear in the published article** if the submission is accepted. Please make sure it is accurate and that any funding	The authors have declared that no competing interests exist.

Powered by Editorial Manager® and ProduXion Manager® from Aries Systems Corporation

sources listed in your Funding Information later in the submission form are also declared in your Financial Disclosure statement.<br><br>View published research articles from *PLOS ONE* for specific examples.<br><br>**NO authors have competing interests**<br><br>Enter: *The authors have declared that no competing interests exist.*<br><br>**Authors with competing interests**<br><br>Enter competing interest details beginning with this statement:<br><br>*I have read the journal's policy and the authors of this manuscript have the following competing interests: [insert competing interests here]*<br><br>* typeset	
**Ethics Statement**<br><br>Enter an ethics statement for this submission. This statement is required if the study involved:<br><br>• Human participants<br>• Human specimens or tissue<br>• Vertebrate animals or cephalopods<br>• Vertebrate embryos or tissues<br>• Field research<br><br>Write "N/A" if the submission does not require an ethics statement.<br><br>General guidance is provided below. Consult the submission guidelines for detailed instructions. **Make sure that all information entered here is included in the Methods section of the manuscript.**	Human Subject Research<br>This study was approved by the Ethics Review Committee for Medical Research of Mie University Hospital (Permit No. 2615).

**Format for specific study types**<br><br>**Human Subject Research (involving human participants and/or tissue)**<br>• Give the name of the institutional review board or ethics committee that approved the study<br>• Include the approval number and/or a statement indicating approval of this research<br>• Indicate the form of consent obtained (written/oral) or the reason that consent was not obtained (e.g. the data were analyzed anonymously)<br><br>**Animal Research (involving vertebrate animals, embryos or tissues)**<br>• Provide the name of the Institutional Animal Care and Use Committee (IACUC) or other relevant ethics board that reviewed the study protocol, and indicate whether they approved this research or granted a formal waiver of ethical approval<br>• Include an approval number if one was obtained<br>• If the study involved *non-human primates*, add *additional details* about animal welfare and steps taken to ameliorate suffering<br>• If anesthesia, euthanasia, or any kind of animal sacrifice is part of the study, include briefly which substances and/or methods were applied<br><br>**Field Research**<br><br>Include the following details if this study involves the collection of plant, animal, or other materials from a natural setting:<br>• Field permit number<br>• Name of the institution or relevant body that granted permission	
**Data Availability**<br><br>Authors are required to make all data underlying the findings described fully available, without restriction, and from the time of publication. PLOS allows rare exceptions to address legal and ethical concerns. See the PLOS Data Policy and FAQ for detailed information.	Yes - all data are fully available without restriction

A Data Availability Statement describing where the data can be found is required at submission. Your answers to this question constitute the Data Availability Statement and **will be published in the article**, if accepted.<br><br>**Important:** Stating 'data available on request from the author' is not sufficient. If your data are only available upon request, select 'No' for the first question and explain your exceptional situation in the text box.<br><br>Do the authors confirm that all data underlying the findings described in their manuscript are fully available without restriction?	
**Describe where the data may be found in full sentences. If you are copying our sample text, replace any instances of XXX with the appropriate details.**<br><br>• If the data are **held or will be held in a public repository**, include URLs, accession numbers or DOIs. If this information will only be available after acceptance, indicate this by ticking the box below. For example: *All XXX files are available from the XXX database (accession number(s) XXX, XXX.*).<br>• If the data are all contained **within the manuscript and/or Supporting Information files**, enter the following: *All relevant data are within the manuscript and its Supporting Information files.*<br>• If neither of these applies but you are able to provide **details of access elsewhere**, with or without limitations, please do so. For example:<br><br>*Data cannot be shared publicly because of [XXX]. Data are available from the XXX Institutional Data Access / Ethics Committee (contact via XXX) for researchers who meet the criteria for access to confidential data.*<br><br>*The data underlying the results presented in the study are available from (include the name of the third party*	All relevant data are within the manuscript and its Supporting Information files.

and contact information or URL).<br>• This text is appropriate if the data are owned by a third party and authors do not have permission to share the data.<br><br>* typeset	
Additional data availability information:	Tick here if the URLs/accession numbers/DOIs will be available only after acceptance of the manuscript for publication so that we can ensure their inclusion before publication.

Powered by Editorial Manager® and ProduXion Manager® from Aries Systems Corporation

Manuscript
Click here to access/download;Manuscript;manuscript.docx

Abstract

The aim of this study was to investigate the usefulness of interim F-18 fluorodeoxyglucose positron emission tomography-computed tomography (FDG PET/CT) during definitive radiation therapy as a prognostic indicator of disease recurrence in patients with hypopharyngeal squamous cell carcinoma. This prospective analysis included 35 patients newly diagnosed with hypopharyngeal squamous cell carcinoma who received platinum-based chemoradiation therapy and underwent pretreatment FDG PET/CT (prePET) and interim FDG PET/CT (iPET) at cumulative RT doses of 36.0–45.0 Gy. The maximum standardized uptake value (SUVmax), metabolic tumor volume, and total lesion glycolysis of the primary tumor and combined total lymph nodes for both prePET and iPET were analyzed, and their percentage reductions in iPET were calculated. The optimal cutoff values of metabolic parameters were derived from receiver operating characteristic curves. Outcomes were compared between patients with metabolic parameters above/below the respective cutoff values. Twelve (34%) patients exhibited disease recurrence during a median follow-up of 4.3 years. Univariate Cox regression analysis revealed that the reduction ratio of the SUVmax of the primary tumor (ΔSUVp; hazard ratio, 7.685; $p = 0.008$) was a significant predictor of disease recurrence. Kaplan–Meier curve analysis revealed that a

smaller ΔSUVp was associated with worse progression-free survival (log-rank, $p = 0.002$). Metabolic parameters measured via iPET may be useful predictors of disease recurrence in patients with hypopharyngeal squamous cell carcinoma treated with chemoradiation therapy. In this study, ΔSUVp was the best such prognostic indicator.

Introduction

Satisfactory results have been reported with the use of concomitant chemoradiation therapy for locally advanced hypopharyngeal cancer to preserve organ function or unresectable disease [1]. However, these treatment regimens are associated with severe late toxicity, such as pharyngeal/swallowing disturbance, which can have a lasting impact on the patient's quality of life [2]. Locoregional recurrence and distant metastasis often occur, and the possibility of successful salvage treatment for recurrent disease is low. Therefore, new biological parameters should be identified to support risk stratification and individualization of treatment for the minimization of toxicity while maintaining therapeutic efficacy. The early identification of such parameters may improve patient outcomes.

F-18 fluorodeoxyglucose (FDG) positron emission tomography (PET)-computed tomography (CT) is useful for staging and radiation therapy (RT) planning for head and neck cancers; moreover, some of its metabolic parameters are known to have prognostic value. The maximum

standardized uptake value (SUVmax), metabolic tumor volume (MTV), and total lesion glycolysis (TLG) are often used as PET metabolic parameters. These parameters are prognostic indicators for patients with locally advanced head and neck squamous cell carcinoma (HNSCC) [3-7], and the prognostic value of FDG PET/CT during RT has been reported [8-11]. However, those reports pertain to a variety of head and neck sites, which may limit the generalizability of their data as tumors from different sites behave differently from both a clinical and a biological perspective. Therefore, the independent evaluation of patients with hypopharyngeal cancer is warranted.

The aim of this study was to investigate the usefulness of interim FDG PET/CT (iPET), performed at a cumulative RT dose ranging from 36.0 to 45.0 Gy of primary RT, as a prognostic indicator of disease recurrence in patients with hypopharyngeal squamous cell carcinoma. In particular, we evaluated whether the residual metabolic tumor burden, determined via the SUVmax, MTV, and TLG, correlated with disease recurrence and whether the metabolic response, assessed via the reduction ratio of these three metabolic parameters, also predicts disease recurrence.

Materials and methods

Study design

This was a prospective cohort study of patients undergoing iPET during chemoradiotherapy for hypopharyngeal cancer. The eligibility criteria were newly diagnosed hypopharyngeal squamous cell carcinoma. Patients with metastatic disease, any other active malignancy, or uncontrolled diabetes were excluded.

Procedures

Pretreatment and interim FDG PET/CT images of patients with hypopharyngeal squamous cell carcinoma who received definitive RT with platinum-based chemotherapy were prospectively evaluated. RT was administered to the primary and neck regions once daily using 6-MV photons. The total irradiation dose of 70.2 Gy was delivered in daily fractions of 1.8 Gy over 8 weeks. After 41.4 Gy was administered, the clinical target volume was reduced to encompass only the primary region and involved neck nodes.

PET/CT scanning technique

Pretreatment PET-CT was taken before treatment, and iPET were performed at cumulative RT doses of 36.0–45.0 Gy. Patients fasted for at least 6 h before the PET examinations, and their blood glucose levels were confirmed to be < 150 mg/dL. FDG PET/CT commenced 60 min after injection of FDG (3.7 MBq/kg body weight) by using the GE Discovery PET/CT 690

system (GE Healthcare, Milwaukee, WI). The PET images covered the area from the skull up to the mid-thighs. The head–neck PET image was acquired for 6 min per bed position in the list mode. The PET images were reconstructed with three-dimensional ordered-subset expectation maximization (2 iterations, 24 subsets, and a 6-mm Gaussian filter) with time-of-flight and point-spread-function modeling and CT attenuation correction. The parameters of CT imaging for attenuation correction included a rotation time of 0.4 s, noise index of 12, tube voltage of 120 kV, and automatic exposure control (tube current range, 10–300 mA). All the PET images were reconstructed using a matrix of 192 × 192 pixels and a slice thickness of 3.27 mm.

FDG-PET image interpretation and metabolic parameter measurement

All the FDG-PET images were analyzed by two experienced radiation oncologists. PET metrics were acquired using imaging software (SYNAPSE SAI Viewer Version 2.0; FUJIFILM, Tokyo, Japan). The SUV_{max} was derived by selecting the area with the most avid uptake at the primary tumor (PT) or nodal sites. The MTV was derived by applying a fixed standardized uptake value (SUV) threshold of 2.5 as the lowest limit of the segmentation criteria. TLG was calculated by multiplying the MTV by the mean standardized uptake value (SUV_{mean}). A fixed SUV threshold of 2.5 was chosen as the segmentation algorithm for the calculation of the MTV

and TLG, consistent with other studies and meta-analyses [3, 7, 12]. The automatically derived, computer-assisted contours and regions of interest were verified on three cross-sectional images (axial, coronal, and sagittal) to ensure that the PT and nodal sites were accurately included and adjacent normal structures excluded. The SUV_{max}, SUV_{mean}, MTV, and TLG were derived for the combined PT and total lymph nodes (PTN) from both pretreatment FDG PET/CT (prePET) and iPET. The reduction ratios of the SUVmax (ΔSUV), MTV (ΔMTV), and TLG (ΔTLG) between the two images were calculated. For example, ΔSUV was calculated using the following equation: ΔSUV =1 – (interim SUVmax / pretreatment SUVmax).

Statistical analysis

The patients were observed for at least 2 years. Physical and endoscopic examinations were performed every 4–8 weeks for at least 2 years after treatment, and diagnostic imaging was performed every 3–6 months. The primary endpoint was progression-free survival (PFS). The secondary endpoint was overall survival (OS). The follow-up duration was calculated from the first day of RT to the date of death or last follow-up visit. PFS was defined as the time from the date of the first RT to the date of death or locoregional or distant recurrence. OS was defined as the time from the date of the first RT to the date of death or the last follow-up. Locoregional recurrences included both recurrences in the original PT region and nodal recurrences within the

region of the external beam radiation fields. Distant metastases were defined as recurrences outside the external beam radiation field. A diagnosis of recurrent tumor or distant metastasis was based on either a positive biopsy result or unequivocal clinical or radiographic evidence of progression. The PFS and OS were estimated using Kaplan–Meier curve analysis and compared using the log-rank test.

FDG PET/CT parameters were compared between the disease-recurrence and non-disease-recurrence groups by using the Mann–Whitney U test. Receiver operating characteristic (ROC) curve analysis was performed to determine the effectiveness of the parameters in predicting disease recurrence. The areas under the ROC curves (AUCs) were calculated, and the optimal cutoff values were determined as the threshold that maximized the Youden index. For univariate analysis, Cox regression analysis was used to identify independent prognostic factors for PFS and OS. Interobserver reliability for the measurement of FDG PET/CT parameters was assessed with the intraclass correlation coefficient (ICC). A two-sided *p*-value less than 0.05 was considered statistically significant. All the analyses were performed using IBM SPSS Statistics for Windows version 23.0 (IBM Corp., Armonk, NY).

Ethics statement

Participants were prospectively recruited between October 10, 2013, and March 31, 2020, at Mie University Hospital. All participants provided written informed consent prior to any study-specific procedure. The study protocol, consent documents, and all amendments were approved by the Ethics Review Committee for Medical Research of Mie University Hospital (Permit No. 2615), and the approval covered the entire recruitment period. Minors were not included.

Results

A total of 35 patients were included in the study from October 10, 2013, to March 31, 2020. The median age at diagnosis was 65 (range, 46–81) years, and 33 patients (94%) were men. Most of the patients (97%) presented with locally advanced disease. T3 and T4 disease comprised 51% of the study population, whereas 69% of the patients had N2 or N3 disease. The most common subsite was the pyriform sinus, which comprised 69% of the study population. RT was completed in all 35 cases. The patient characteristics are summarized in Table 1.

Table 1. Patient characteristics

**Characteristics**		**No. of patients (%)**
		Total (n = 35)

**Follow-up time**	Median (range)	52 (4–64) months
**Age**	Median (range)	65 (46–81) years
**Sex**	Male	33 (94)
	Female	2 (6)
**ECOG PS**	0	19 (54)
	1	14 (40)
	2	2 (6)
**Smoking history**	Current smoker	15 (43)
	Former smoker	20 (57)
**Primary tumor subsite**	Pyriform sinus	24 (69)
	Posterior pharyngeal wall	7 (20)
	Postcricoid region	4 (11)
**T classification**	1	2 (6)
	2	15 (43)
	3	10 (29)
	4	8 (23)
**N classification**	0	3 (9)
	1	8 (23)

	2	22 (63)
	3	2 (6)
**Stage (7th UICC TNM classification)**	Ⅱ	1 (3)
	Ⅲ	8 (23)
	Ⅳ	26 (74)
**Radiation dose**	70.2 Gy in 39 fractions	35 (100)
**Chemotherapeutic regimen**	5-Fluorouracil + cisplatin	14 (40)
	Cisplatin alone	14 (40)
	5-Fluorouracil + carboplatin	4 (11)
	Carboplatin alone	3 (9)

Abbreviations: ECOG PS: Eastern Cooperative Oncology Group performance status, UICC: Union for International Cancer Control, TNM: tumor, nodes, metastases.

The median metabolic values of the PT for prePET vs. iPET were as follows: SUVmax: 14.99 (range, 3.19–25.99) vs. 4.31 (1.55–10.90); MTV: 13.45 (0.20–45.80) vs. 2.65 (0–23.25) cm^3; and TLG: 78.60 (0.70–328.90) vs. 9.3 (0–110.35). The median metabolic values of the index node for prePET vs. iPET were as follows: SUVmax: 7.65 (range, 0–29.32) vs. 2.64 (0–12.64); MTV: 1.60 (0–89.05) vs. 0.10 (0–85.20) cm^3; and TLG: 6.60 (0–685.55) vs. 0.20 (0–525.05).

At the time of analysis, 26 patients (74%) were alive and 23 (66%) were disease-free, whereas 12 (34%) had treatment failure (9 with locoregional failure, 7 with distant failure, and 4 with both locoregional and distant failure). The median follow-up of all the patients was 52 (range, 4–64; mean, 46) months, whereas that of the survivors was 61 (range, 30–64; mean, 55) months. Overall, 9 of the 35 patients (26%) died during the follow-up period, 7 of the tumor and 2 of other causes. All the recurrence-free cases were followed for at least 2 years. After chemoradiation therapy, 12 patients exhibited disease recurrence, whereas 23 did not. Table 2 summarizes the results of the association between PET parameters and disease recurrence. Significant differences were observed in the residual metabolic burden of iPET and the reduction ratios between the disease- and non-disease-recurrence groups, whereas no significant differences were observed for the prePET parameters.

Table 2. Association between PET parameters and disease recurrence for the 35 patients

**Parameter**	**Disease recurrence (+) (n = 12)**	**Disease recurrence (-) (n = 23)**	***P*-value**
	**Median (IQR)**	**Median (IQR)**	
**preSUVpmax**	14.1 (9.7–17.9)	15.2 (11.4–18.3)	0.851
**preMTVp (cm^3)**	18.6 (6.7–28.4)	12.7 (5.4–28.6)	0.572

**preTLGp**	121.5 (28.8–186.9)	68.9 (29.7–164.9)	0.668
**preMTVptn (cm^3)**	36.1 (11.7–43.8)	15.3 (8.9–34.8)	0.161
**preTLGptn**	214.4 (65.0–293.7)	84.5 (41.7–201.1)	0.172
**iSUVpmax**	6.1 (4.9–8.0)	4.0 (2.6–5.1)	0.004
**iMTVp (cm^3)**	4.9 (1.6–8.3)	2.0 (0.1–4.5)	0.028
**iTLGp**	17.8 (5.8–35.8)	6.1 (0.3–16.6)	0.028
**iMTVptn (cm^3)**	8.3 (3.4–13.6)	2.3 (0.2–6.3)	0.016
**iTLGptn**	30.3 (13.4–55.0)	9.1 (0.6–20.2)	0.011
**ΔSUVp**	0.556 (0.443–0.670)	0.750 (0.641–0.807)	0.002
**ΔMTVp**	0.644 (0.557–0.848)	0.862 (0.717–0.981)	0.026
**ΔTLGp**	0.805 (0.712–0.907)	0.930 (0.851–0.988)	0.017
**ΔMTVptn**	0.720 (0.607–0.767)	0.853 (0.745–0.965)	0.011
**ΔTLGptn**	0.817 (0.732–0.861)	0.909 (0.856–0.981)	0.005

Abbreviations: PET: positron emission tomography, IQR: interquartile range, SUVpmax: maximum standardized uptake value of primary tumor, MTV: metabolic tumor volume, TLG: total lesion glycolysis, preSUVpmax: pretreatment SUVpmax, preMTVp: pretreatment MTV of primary tumor, preTLGp: pretreatment TLG of primary tumor, preMTVptn: pretreatment MTV of combined primary tumor and total lymph nodes, preTLGptn: pretreatment TLG of combined

primary tumor and total lymph nodes, iSUVpmax: interim SUVpmax, iMTVp: interim MTV of primary tumor, iTLGp: interim TLG of primary tumor, iMTVptn: interim MTV of combined primary tumor and total lymph nodes, iTLGptn: interim TLG of combined primary tumor and total lymph nodes, ΔSUVp: reduction ratio of SUVpmax, ΔMTVp: reduction ratio of MTV of primary tumor, ΔTLGp: reduction ratio of TLG of primary tumor, ΔMTVptn: reduction ratio of MTV of combined primary tumor and total lymph nodes, ΔTLGptn: reduction ratio of TLG of combined primary tumor and total lymph nodes.

Table 3. presents the optimal cutoff values derived from the ROC analysis. The cutoff values of the PT for iPET were as follows: SUVmax: 4.85 (AUC = 0.80, p = 0.005), MTV: 4.05 cm3 (AUC = 0.73, p = 0.029), and TLG: 12.35 (AUC = 0.73, p = 0.029). The cutoff values of the PTN for iPET were as follows: MTV: 10.30 cm3 (AUC = 0.75, p = 0.017) and TLG: 27.75 (AUC = 0.76, p = 0.012). The cutoff values of the ΔSUVmax of the PT (ΔSUVp) were as follows: ΔSUVmax: 0.69 (AUC = 0.81, p = 0.003), ΔMTV: 0.77 (AUC = 0.73, p = 0.026), and ΔTLG: 0.84 (AUC = 0.75, p = 0.018). The cutoff values of the ΔMTV of the PTN were as follows: ΔMTV: 0.77 (AUC = 0.76, p = 0.012) and ΔTLG: 0.86 (AUC = 0.79, p = 0.006).

Table 3. Cutoff values for the prediction of treatment outcomes using receiver operating

characteristic analysis

**Parameter**	**Cutoff value**	**AUC (95% CI)**	**Sensitivity**	**Specificity**	***P*-value**
**iSUVpmax**	4.85	0.80 (0.63–0.95)	0.83	0.70	0.005
**iMTVp (cm^3)**	4.05	0.73 (0.55–0.90)	0.67	0.74	0.029
**iTLGp**	12.35	0.73 (0.55–0.90)	0.75	0.70	0.029
**iMTVptn (cm^3)**	10.30	0.75 (0.57–0.93)	0.50	0.96	0.017
**iTLGptn**	27.75	0.76 (0.59–0.94)	0.58	0.91	0.012
**ΔSUVp**	0.69	0.81 (0.67–0.95)	0.92	0.65	0.003
**ΔMTVp**	0.77	0.73 (0.56–0.91)	0.75	0.74	0.026
**ΔTLGp**	0.84	0.75 (0.58–0.91)	0.67	0.78	0.018
**ΔMTVptn**	0.77	0.76 (0.59–0.93)	0.83	0.74	0.012
**ΔTLGptn**	0.86	0.79 (0.62–0.95)	0.83	0.74	0.006

Abbreviations: AUC: area under the receiver operating characteristic curve, CI: confidence interval, SUVpmax: maximum standardized uptake value of primary tumor, MTV: metabolic tumor volume, TLG: total lesion glycolysis, iSUVpmax: interim SUVpmax, iMTVp: interim MTV of primary tumor, iTLGp: interim TLG of primary tumor, iMTVptn: interim MTV of combined primary tumor and total lymph nodes, iTLGptn: interim TLG of combined primary tumor and total lymph nodes, ΔSUVp: reduction ratio of SUVpmax, ΔMTVp: reduction ratio of

MTV of primary tumor, ΔTLGp: reduction ratio of TLG of primary tumor, ΔMTVptn: reduction ratio of MTV of combined primary tumor and total lymph nodes, ΔTLGptn: reduction ratio of TLG of combined primary tumor and total lymph nodes.

Table 4 summarizes the results of the univariate analysis. The residual metabolic burdens on iPET and the reduction ratio were significantly associated with disease recurrence. ΔSUVp > 0.69 was associated with a favorable PFS (hazard ratio [HR], 7.685; 95% confidence interval [CI], 1.706–34.626; p = 0.008). An interim SUVmax of the PT ≤ 4.85 was associated with a favorable OS (HR, 12.366; 95% CI, 1.525–100.3; p = 0.019). Kaplan–Meier curve analysis (Fig 1) demonstrated that patients with a ΔSUVp ≤ 0.69 had significantly lower rates of PFS (3-year rate, 37% vs. 94%; p = 0.002; Fig A) than those with ΔSUVp > 0.69. ΔSUVp ≤ 0.69 was associated with a worse OS (62% vs. 94%; p = 0.064; Fig B) than ΔSUVp > 0.69.

Fig 1. Kaplan–Meier curves stratified by ΔSUVp cutoff (0.69). (A) PFS by ΔSUVp, (B) OS by ΔSUVp.

(A) Kaplan–Meier curves of patients stratified according to the cutoff value (0.69) of ΔSUVp for the prediction of progression-free survival. (B) Kaplan–Meier curves of patients stratified

according to the cutoff value (0.69) of ΔSUVp for the prediction of overall survival. Patients with a ΔSUVp ≤ 0.69 had significantly worse PFS than those with a ΔSUVp > 0.69 (p = 0.002).

Table 4. Univariate analysis for progression-free survival and overall survival for all patients

**Parameter**	**Progression-free survival**			**Overall survival**		
	**HR**	**95% CI**	***P*-value**	**HR**	**95% CI**	***P*-value**
**iSUVpmax (≥ 4.85 vs. < 4.85)**	6.16	1.70–22.42	0.006	12.37	1.53–100.3	0.019
**iMTVp (≥ 4.05 vs. < 4.05)**	2.91	1.00–8.44	0.05	2.39	0.64–8.91	0.196
**iTLGp (≥ 12.35 vs. < 12.35)**	3.29	1.09–9.91	0.034	3.21	0.80–12.94	0.101
**iMTVptn (≥ 10.30 vs. < 10.30)**	6.92	2.26–21.21	0.001	10.53	2.68–41.42	0.001
**iTLGptn (≥ 27.75 vs. < 27.75)**	5.35	1.80–15.86	0.003	6.38	1.64–24.87	0.008
**ΔSUVp (≤ 0.69 vs. > 0.69)**	7.69	1.71–34.63	0.008	3.97	0.82–19.30	0.087
**ΔMTVp (≤ 0.77 vs. > 0.77)**	3.68	1.23–11.07	0.02	6.53	1.35–31.70	0.02
**ΔTLGp (≤ 0.84 vs. > 0.84)**	3.29	1.13–9.55	0.029	8.81	1.81–42.87	0.007
**ΔMTVptn (≤ 0.77 vs. > 0.77)**	4.58	1.43–14.72	0.011	5.78	1.19–28.04	0.03
**ΔTLGptn (≤ 0.86 vs. > 0.86)**	4.58	1.43–14.72	0.011	5.78	1.19–28.04	0.03

Abbreviations: HR: hazard ratio, CI: confidence interval, SUVpmax: maximum standardized

uptake value of primary tumor, MTV: metabolic tumor volume, TLG: total lesion glycolysis, iSUVpmax: interim SUVpmax, iMTVp: interim MTV of primary tumor, iTLGp: interim TLG of primary tumor, iMTVptn: interim MTV of combined primary tumor and total lymph nodes, iTLGptn: interim TLG of combined primary tumor and total lymph nodes, ΔSUVp: reduction ratio of SUVpmax, ΔMTVp: reduction ratio of MTV of primary tumor, ΔTLGp: the reduction ratio of the TLG of primary tumor, ΔMTVptn: reduction ratio of the MTV of combined primary tumor and total lymph nodes, ΔTLGptn: reduction ratio of TLG of combined primary tumor and total lymph nodes.

High interobserver reliability was observed for all the parameters. The ICCs of the PT for prePET were as follows: SUVmax: 1.000 (95% CI, 1.000–1.000), MTV: 0.999 (95% CI, 0.997–0.999), and TLG: 1.000 (95% CI, 1.000–1.000). The ICCs of the PT for iPET were as follows: MTV: 1.000 (95% CI, 0.999–1.000) and TLG: 1.000 (95% CI, 1.000–1.000). The ICCs of the PTN for prePET were as follows: SUVmax: 1.000 (95% CI, 1.000–1.000), MTV: 0.996 (95% CI, 0.993–0.998), and TLG: 0.999 (95% CI, 0.999–1.000). The ICCs of PTN for iPET were as follows: MTV: 1.000 (95% CI, 1.000–1.000) and TLG: 1.000 (95% CI, 1.000–1.000).

Discussion

To our knowledge, this is the first prospective study in which the prognostic value of FDG PET/CT performed before and during RT was evaluated for hypopharyngeal cancer. This report suggests that the prePET and iPET parameters may be prognostic factors for patients with hypopharyngeal cancer. These metabolic parameters possess the potential to stratify patients into poor and good prognosis. Therefore, iPET parameters may allow for the relatively early detection of treatment-resistant diseases that are not detected via prePET alone, allowing for individually adapted treatment.

Data on the prognostic value of the residual metabolic burden of iPET for HNSCC are limited. In the study by Farrag et al., the SUVmax of PTs at baseline and during the treatment after 47 Gy was associated with OS [13]. Kim et al. reported that the TLG of the PT during RT was the most statistically significant prognostic factor for OS and PFS [11]. In this study, the associations of the reduction ratios of metabolic values and oncological outcomes have not been well studied. Hentschel et al. evaluated iPET in patients with HNSCC and demonstrated that a decrease of 50% or more in SUVmax of the PT from the beginning (0 Gy) to 1 or 2 weeks (10 or 20 Gy) of treatment is a potential prognostic marker for patients with HNSCC [14]. Chen et al. studied the SUVp after a cumulative dose of 40–50 Gy during RT and reported on a correlation between the ΔSUVp and oncological outcomes [8]. However, Min et al. evaluated iPET in the third week of RT and discovered no significant associations between the percentage

reduction of any of the metabolic parameters (ΔSUVmax, ΔMTV, and ΔTLG) and oncological outcomes [12].

In our study, the reduction ratios, rather than the residual metabolic burden, predicted disease recurrence in patients with hypopharyngeal cancer. The ΔSUVp was the best prognostic indicator for disease recurrence. Use of the reduction ratio rather than reliance on an absolute value (the residual metabolic burden) also has the potential benefit of improving the standardization and reproducibility of longitudinal studies. This benefit is attributable to this parameter’s lower susceptibility to treatment-related confounding factors, such as mucositis, and differences in equipment and protocols among hospitals. Therefore, we expect that the selection of optimal ΔSUVp values will minimize inherent and individual site biases.

Our results imply that the ΔSUVp of patients with advanced hypopharyngeal cancer is useful for the identification of patients at high risk of disease recurrence. Patients with a low ΔSUVp may require a more aggressive treatment plan and shorter follow-up intervals after treatment. Further research is also warranted to determine the optimal timing of iPET. Although earlier iPET may be useful in distinguishing metabolic changes from inflammatory changes and aiding early decisions concerning treatment-plan modifications [10], later assessments may also be valid.

Several reports have indicated that dose escalation may improve outcomes in head and neck cancer [15-17]; however, therapy-related complications may be increased by dose escalation owing to the high radiation dose delivered to normal tissues [18]. Therefore, such escalation should be performed by irradiating a small field with a cone-down boost. We confirmed that the MTV was sufficiently reduced at the time of iPET performed at a cumulative RT dose of 36–45 Gy in the current study. Therefore, the volume of normal tissue that receives high doses may be reduced and the treatment effect may improve by optimizing the timing of iPET and considering dose escalation.

A limitation of this study is that it was a single-center study with a small number of patients who had been treated with various chemotherapeutic regimens. In addition, the sample size was too small for multivariate analysis. However, its strengths include its prospective design and relatively homogenous patient population. Another strength of this study is that it dealt only with patients with hypopharyngeal cancer treated with definitive chemoradiation therapy; this is important, as tumors from different sites behave differently from clinical and biological points of view [19]. In accordance with consensus recommendations for the use of FDG-PET [20], all our patients underwent pretreatment and interim imaging via the same FDG-PET protocol and were analyzed using the same software to minimize variability in this study.

Conclusions

The residual volumetric metabolic burden and its percentage reduction derived from iPET are indicated to be useful imaging biomarkers for prediction of the risk of disease recurrence in patients with hypopharyngeal cancer treated with chemoradiation therapy. These biomarkers may play a role in individualized adaptive chemoradiation therapy, particularly with respect to the selection of dose intensification and adjuvant chemotherapy.

Competing interests

The authors report there are no competing interests to declare.

Data availability statement

All relevant data are within the paper and its Supporting Information files.

Author Contributions

Conceptualization: TM, YT, YN.

Methodology: TM, YT.

Investigation: HI, TK, AT, TH.

Data curation: TM, YT, YI.

Formal analysis: TM, YT, YI.

Visualization: TM, YT, YI.

Writing – original draft: TM, YN.

Writing – review & editing: All authors.

Supervision: YN.

Acknowledgments

We would like to thank Editage (www.editage.jp) for English language editing.

References

[1] Pignon JP, Bourhis J, Domenge C, Designé L. Chemotherapy added to locoregional treatment for head and neck squamous cell carcinoma: three meta-analyses of updated individual data. MACH-NC Collaborative Group. Meta-analysis of chemotherapy for head and neck cancers. Lancet. 2000;355:949–55. https://doi.org/10.1016/S0140-6736(00)90011-4.

[2] Machtay M, Moughan J, Farach A, Martin-O'Meara E, Galvin J, Garden AS, et al. Hypopharyngeal dose is associated with severe late toxicity in locally advanced head and neck cancer: An RTOG analysis. Int J Radiat Oncol Biol Phys. 2012;84:983–9. https://doi.org/10.1016/j.ijrobp.2012.03.005.

[3] Pak K, Cheon GJ, Nam HY, Kim SJ, Kang KW, Chung JK, et al. Prognostic value of metabolic tumor volume and total lesion glycolysis in head and neck cancer: A systematic review and meta-analysis. J Nucl Med. 2014;55:884–90. https://doi.org/10.2967/jnumed.113.133801.

[4] Kim JW, Oh JS, Roh JL, Kim JS, Choi SH, Nam SY, et al. Prognostic significance of standardized uptake value and metabolic tumor volume on ^{18}F-FDG PET/CT in oropharyngeal squamous cell carcinoma. Eur J Nucl Med Mol Imaging. 2015;42:1353–61. https://doi.org/10.1007/s00259-015-3051-4.

[5] Cacicedo J, Fernandez I, Del Hoyo O, Navarro A, Gomez-Iturriaga A, Pijoan JI, et al. Prognostic value of maximum standardized uptake measured by pretreatment 18F-FDG PET/CT in locally advanced head and neck squamous cell carcinoma. Clin Transl Oncol. 2017;19:1337–49. https://doi.org/10.1007/s12094-017-1674-6.

[6] Bonomo P, Merlotti A, Olmetto E, Bianchi A, Desideri I, Bacigalupo A, et al. What is the prognostic impact of FDG PET in locally advanced head and neck squamous cell carcinoma treated with concomitant chemo-radiotherapy? A systematic review and meta-analysis. Eur J Nucl Med Mol Imaging. 2018;45:2122–38. https://doi.org/10.1007/s00259-018-4065-5.

[7] Wang L, Bai J, Duan P. Prognostic value of 18F-FDG PET/CT functional parameters in patients with head and neck cancer: a meta-analysis. Nucl Med Commun. 2019;40:361–9. https://doi.org/10.1097/MNM.0000000000000974.

[8] Chen SW, Hsieh TC, Yen KY, Yang SN, Wang YC, Chien CR, et al. Interim FDG PET/CT for predicting outcomes in patients with head and neck cancers. Laryngoscope. 2014;124:2732–8. https://doi.org/10.1002/lary.24826.

[9] Pollom EL, Song J, Durkee BY, Aggarwal S, Bui T, von Eyben R, et al. Prognostic value of midtreatment FDG-PET in oropharyngeal cancer. Head Neck. 2016;38:1472–8. https://doi.org/10.1002/hed.24454.

[10] Garibaldi C, Ronchi S, Cremonesi M, Gilardi L, Travaini L, Ferrari M, et al. Interim (18)F-FDG PET/CT during chemoradiation therapy in the management of head and neck cancer patients: A systematic review. Int J Radiat Oncol Biol Phys. 2017;98:555–73. https://doi.org/10.1016/j.ijrobp.2017.02.217.

[11] Kim S, Oh S, Kim JS, Kim YK, Kim KH, Oh DH, et al. Prognostic value of FDG PET/CT during radiotherapy in patients with head and neck cancers. Radiat Oncol J. 2018;36:95–102. https://doi.org/10.3857/roj.2017.00577.

[12] Min M, Lin P, Lee MT, Shon IH, Lin M, Forstner D, et al. Prognostic role of metabolic parameters of (18)F-FDG PET-CT performed during radiation therapy in locally advanced head and neck squamous cell carcinoma. Eur J Nucl Med Mol Imaging. 2015;42:1984–94. https://doi.org/10.1007/s00259-015-3104-8.

[13] Farrag A, Ceulemans G, Voordeckers M, Everaert H, Storme G. Can 18F-FDG-PET response during radiotherapy be used as a predictive factor for the outcome of patients with head and neck cancer patients? Nucl Med Commun. 2010;31:495–501. https://doi.org/10.1097/MNM.0b013e3283334e2b.

[14] Hentschel M, Appold S, Schreiber A, Abolmaali N, Abramyuk A, Dörr W, et al. Early FDG PET at 10 or 20 Gy under chemoradiotherapy is prognostic for locoregional control and overall survival in patients with head and neck cancer. Eur J Nucl Med Mol Imaging. 2011;38:1203–11. https://doi.org/10.1007/s00259-011-1759-3.

[15] Atwell D, Elks J, Cahill K, Hearn N, Vignarajah D, Lagopoulos J, et al. A review of modern radiation therapy dose escalation in locally advanced head and neck cancer. Clin Oncol (R Coll Radiol). 2020;32:330–41. https://doi.org/10.1016/j.clon.2019.12.004.

[16] Fu S, Li Y, Han Y, Wang H, Chen Y, Yan O, et al. Diffusion-weighted magnetic resonance imaging-guided dose painting in patients with locoregionally advanced nasopharyngeal carcinoma treated with induction chemotherapy plus concurrent chemoradiotherapy: A randomized, controlled clinical trial. Int J Radiat Oncol Biol Phys. 2022;113:101–13. https://doi.org/10.1016/j.ijrobp.2021.12.175.

[17] Wang J, Zheng J, Tang T, Zhu F, Yao Y, Xu J, et al. A randomized pilot trial comparing position emission tomography (PET)-guided dose escalation radiotherapy to conventional radiotherapy in chemoradiotherapy treatment of locally advanced nasopharyngeal carcinoma. PLoS One. 2015;10:e0124018. https://doi.org/10.1371/journal.pone.0124018.

[18] Bakst RL, Lee N, Pfister DG, Zelefsky MJ, Hunt MA, Kraus DH, et al. Hypofractionated dose-painting intensity modulated radiation therapy with chemotherapy for nasopharyngeal carcinoma: a prospective trial. Int J Radiat Oncol Biol Phys. 2011;80:148–53. https://doi.org/10.1016/j.ijrobp.2010.01.026.

[19] De Cecco L, Nicolau M, Giannoccaro M, Daidone MG, Bossi P, Locati L, et al. Head and neck cancer subtypes with biological and clinical relevance: meta-analysis of gene-expression data. Oncotarget. 2015;6:9627–42. https://doi.org/10.18632/oncotarget.3301.

[20] Shankar LK, Hoffman JM, Bacharach S, Graham MM, Karp J, Lammertsma AA, et al. Consensus recommendations for the use of 18F-FDG PET as an indicator of therapeutic response in patients in National Cancer Institute Trials. J Nucl Med. 2006;47:1059–66.

 Click here to access/download;Figure;Fig1.tif

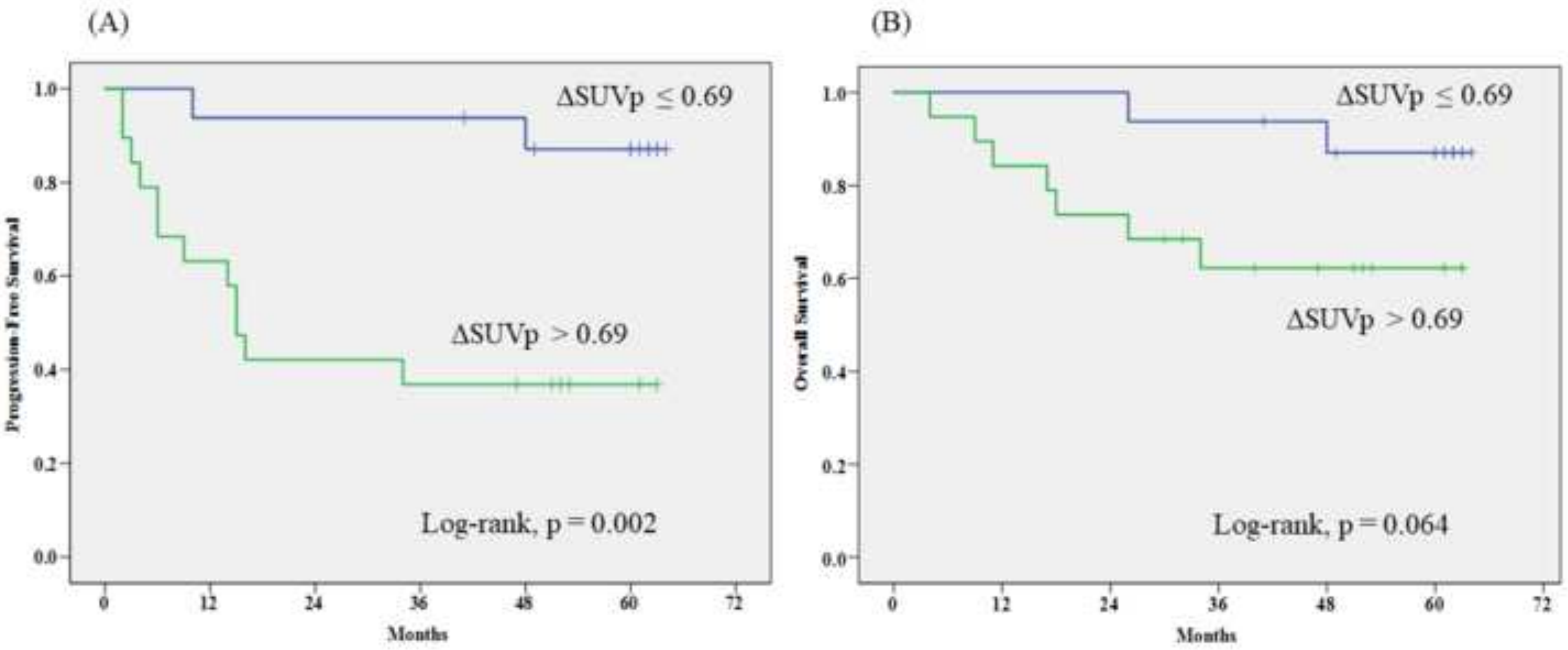

Supplement: Attachment — Submitted filename: PONE-D-25-48920_reviewer_RJD.pdf [file pone.0341189.s002.pdf]
